# Editorial: Trained Immunity-Based Vaccines

**DOI:** 10.3389/fimmu.2021.716296

**Published:** 2021-06-24

**Authors:** Jose Luis Subiza, Oscar Palomares, Isabella Quinti, Silvia Sánchez-Ramón

**Affiliations:** ^1^ Inmunotek SL, Alcalá de Henares, Spain; ^2^ Department of Biochemistry and Molecular Biology, Faculty of Chemistry, Complutense University of Madrid, Madrid, Spain; ^3^ Department of Clinical and Molecular Medicine, Faculty of Medicine and Psychology, Sapienza University, Rome, Italy; ^4^ Department of Clinical Immunology, San Carlos University Clinical Hospital, Madrid, Spain

**Keywords:** trained immunity, vaccine, immunostimulant, adjuvant, infection, TIbV

Trained immunity is defined as a type of memory of the innate immune system by which innate immune cells undergo a long-term adaptation, largely dependent on persistent epigenetic modifications and metabolic reprogramming of these cells (1). Myeloid cells can be trained with a variety of stimuli (typically of microbial origin) that improve their responsiveness to second stimuli (same or unrelated) in a fairly stable manner. The mechanistic basis of trained immunity and the diversity of trained cells behaviors may have important implications not only for innate but also for adaptive immunity and, therefore, for a response to vaccines or for their design (2).

The emerging concept of trained immunity-based vaccines (TIbV) (3) challenges that of traditional vaccines in several intriguing ways, whose clinical and immunological implications deserve further exploration: i) TIbV may act beyond their antigenic formulation, providing non-specific protection against different pathogens based on trained (innate) immune cells; ii) TIbV have self-adjuvant properties enhancing adaptive immune responses to their own antigens, but also to bystander antigens. Their potential use outside infectious prevention is opening new immunotherapy approaches in other conditions like cancer (4) or allergies (5).

This special issue has gathered a number of latest resonance original research studies and reviews from authors working in different areas related to this topic.

Trained immunity depends on the presence of trained innate cells. Although myeloid cells are considered in general short-lived, trained immunity may last a quite long time (from several months to over a year). This may be due to the presence of long-lived trained macrophages and/or myeloid precursors in the bone marrow. In this issue, Chen and Ozato review how hematopoietic stem/progenitor cells can acquire epigenetic memory upon pathogen exposure and the soluble mediators, e.g. interferons, involved in memory formation within the bone marrow.

The heterologous protection associated with BCG vaccination is one of the best-studied examples linking non-specific protection and trained immunity in a clinical setting. Gonzalez-Perez et al. have reviewed the potential use of BCG vaccine in COVID-19 pandemics. Several clinical trials have been initiated to address whether BCG vaccination confers non-specific protection against SARS-CoV-2, and/or associated infections. This trained immunity-based approach could make it possible to increase the resistance of vulnerable subjects in the context of viral outbreaks before a specific vaccine is ready (6). Indeed, even if such a vaccine exists, outbreaks can eventually appear in a vaccinated population by a number of factors reviewed by Connell et al., exemplified by recent outbreaks of mumps in MMR-vaccinated subjects.

The potential of BCG as training inducer can be harnessed to use it together with specific antigens to simultaneously induce specific (adaptive) and non-specific innate immunity. In this line, Covián et al. have reviewed an interesting novel approach using recombinant BCGs expressing antigens from the syncytial virus and metapneumovirus, i.e., as a canonical TIbV. An alternative possibility is to split the TIbV in two separated elements, i.e., the trained immunity-inducer as adjuvant and the nominal antigen (3). This approach has been tested by Paris et al. in veterinary medicine using a pretreatment with β-glucan (from *Euglena gracilis*) to enhance the specific immune response to a rabies vaccine administered at the same time or one month later. Thus, training may be used to optimize vaccine immunization strategies. This has been pointed out in the review by Palgen et al., addressing how myeloid innate cells sense and respond differently to a first and a second dose of vaccine and how trained innate cells can be harnessed to optimize the response to vaccination.

Most described trained immunity-inducers are microbial-derived products that stimulate innate immune cells through different PRRs (pattern recognition receptors). Either the nucleotide-binding oligomerization domain (NOD)-Leucin Rich Repeats (LRR)-containing receptors (NLR) or the C-type lectin receptors (CLRs) have been involved, for example NOD2 (BCG) or Dectin-1 (yeast β-glucans). The involvement of Toll-like receptors (TLRs) as trained-immunity inducers seems less clear (7). TLR ligands, however, are used as adjuvants in different vaccine formulations and shown to confer non-specific anti-infectious protection in different settings. The interplay of TLR agonists with the phenomenon of trained immunity has been reviewed by Owen et al. with an overview of TLR signaling as potential activating mechanisms of trained immunity. Such an interplay may be supported by the fact that the pro-inflammatory cytokine profile (TNF-α, IL-6, and IL-1β), well-known hallmarks of trained immunity, is severely affected in TLR4–/– mice, as described by Sánchez-Tarjuelo et al. This experimental model discloses an impaired innate immune response to a challenge with *S. pneumoniae* indicating the critical involvement of TLR4 in the resistance against these Gram-positive bacteria. On the other hand, Vázquez et al. show how mesenchymal stem cells are able to uptake, process and retain a reservoir of the TLR ligands derived from inactivated bacteria (MV130), which can subsequently be transferred to dendritic cells. MV130 is a mucosal polybacterial preparation that induces trained immunity and prevents viral wheezing attacks in young children (8) and used in a proof of concept study in patients with hematological malignancies, showing beneficial effects in terms of infections incidence (9). The effect of the combination of a similar polybacterial preparation (MV140) used to prevent urinary tract infections with *Candida albicans* (V132) has been studied by Martín-Cruz et al. The authors show how this combination (MV140/V132) promotes metabolic and epigenetic reprogramming in human DCs, which are key molecular mechanisms involved in the induction of trained immunity, while enhancing specific responses to their nominal antigens.

Vaccine formulations based on DCs loaded with recombinant proteins or mRNA derived from *Listeria monocytogenes* Glyceraldehyde-3-Phosphate Dehydrogenase (LM-GAPDH), have been studied by Teran-Navarro et al. In contrast to mRNA, the recombinant proteins induce non-specific DC activation and show higher immunogenicity. The interesting point of this approach is that LM-GADPH is highly cross-reactive with that derived from other pathogens, and vaccinated mice are protected not only to a challenge with *Listeria monocytogenes*, but also to *Mycobacterium marinum* or *Streptococcus pneumoniae*.

In the field of cancer immunotherapy, Zhang et al. have reviewed the functionality of DCs in the tumor microenvironment of ovarian cancer, the role of tumor suppressor signals, and the use of tumor antigen-loaded DCs as cancer vaccines. In their comprehensive review, the authors analyze the different possibilities of these vaccines, alone or in combination with other immunotherapies, as well as possible useful biomarkers.

Finally, Pontigo et al. describe a vaccine candidate inducing an effective protection of Atlantic salmon against *Piscirickettsia salmonis* infection in correlation with the production of Piscirickettsia-specific IgM antibodies and pro-inflammatory cytokines (IL-1β and TNF-α) in contrast to other prototypes that stimulate only innate immunity.

In summary, this timely Research Topic showcases the latest findings and new insights into the potential of trained immunity in vaccine development. TIbV are conceptually novel vaccines that might well confer antigen specific but also antigen non-specific resistance to unrelated pathogens as described for immunostimulants (**Figure 1**). This last aspect highlights the need to review the clinical evaluation of TIbV beyond the specific responses of conventional anti-infectious vaccines. A better understanding of the interaction of different trained immunity inducers, at the molecular and cellular level, could well pave the way for a better design of TIbV and their clinical applications after adequate translational research.

**Figure 1:**
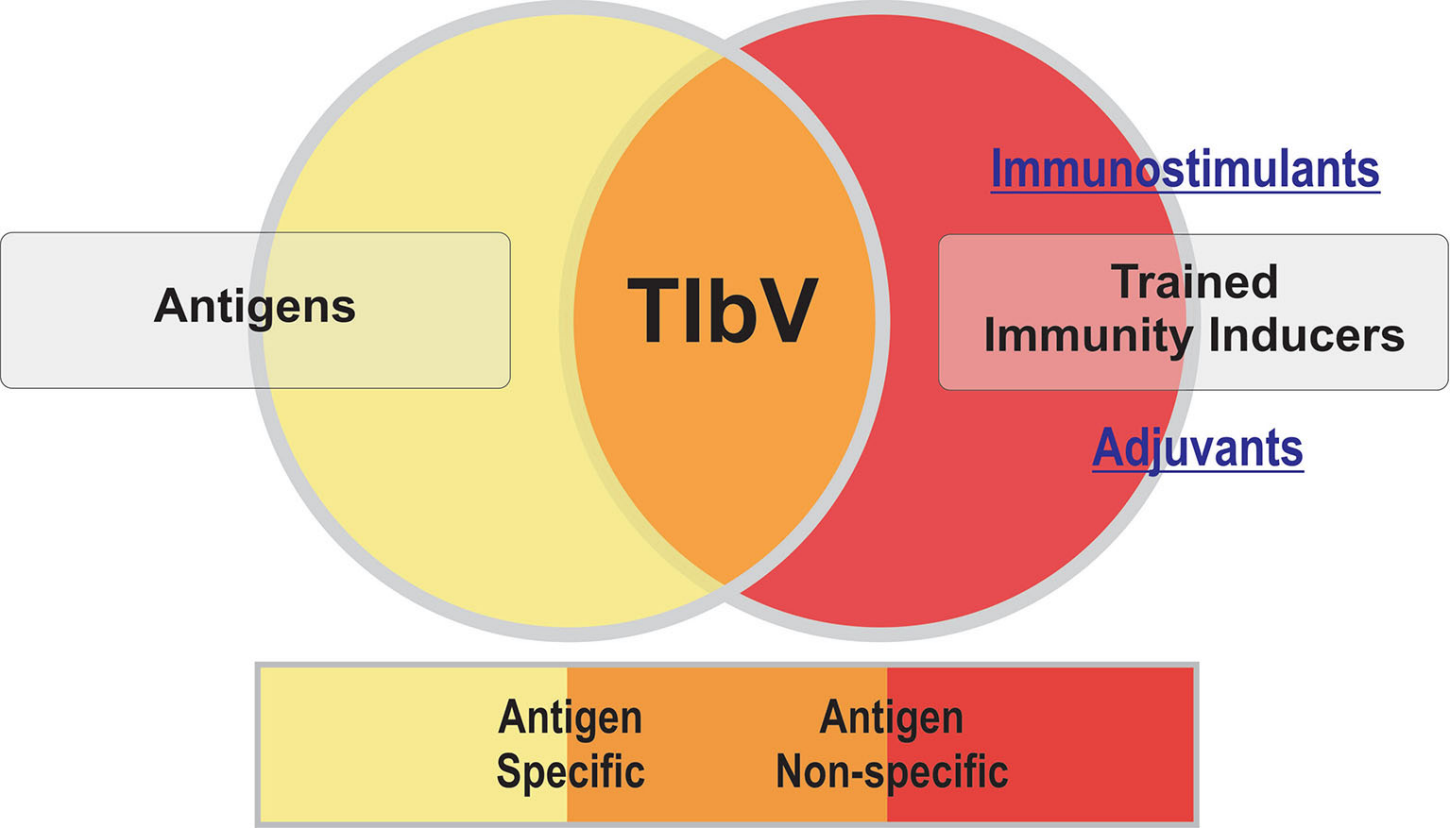
Trained immunity-based vaccines (TIbV) features.

## Author Contributions

JS wrote the manuscript, together with OP and SS-R. IQ read it and approved it for publication. All authors contributed to the article and approved the submitted version.

## Conflict of Interest

JS is the founder and CEO-President of Inmunotek.

The remaining authors declare that the research was conducted in the absence of any commercial or financial relationships that could be construed as a potential conflict of interest.
